# Experimental Chemistry and Structural Stability of AlNb_3_ Enabled by Antisite Defects Formation

**DOI:** 10.3390/ma12071104

**Published:** 2019-04-03

**Authors:** Nikola Koutná, Petra Erdely, Siegfried Zöhrer, Robert Franz, Yong Du, Shuhong Liu, Paul H. Mayrhofer, David Holec

**Affiliations:** 1 Institute of Materials Science and Technology, TU Wien, Getreidemarkt 9, A-1060 Vienna, Austria; 2 Department of Condensed Matter Physics, Faculty of Science, Masaryk University, Kotlářská 2, CZ-611 37 Brno, Czech Republic; 3 Department of Materials Science, Montanuniversität Leoben, Franz-Josef-Strasse 18, A-8700 Leoben, Austria; 4 State Key Laboratory of Powder Metallurgy, Central South University, Changsha 410083, China

**Keywords:** ab initio, CALPHAD, phase stability, phonons, point defects, AlNb_3_

## Abstract

First-principles evolutionary algorithms are employed to shed light on the phase stability of Al–Nb intermetallics. While the tetragonal Al_3_Nb and AlNb_2_ structures are correctly identified as stable, the experimentally reported Laves phase of AlNb_3_ yields soft phonon modes implying its dynamical instability at 0 K. The soft phonon modes do not disappear even upon elevating the temperature in the simulation up to 1500 K. X-Ray diffraction patterns recorded for our powder-metallurgically produced arc cathodes, however, clearly show that the AlNb_3_ phase exists. We propose that AlNb_3_ is dynamically stabilised by ordered antisite defects at the Al sublattice, leading also to a shift of the Nb content from 75 to ∼81 at.%. Unlike the defect-free AlNb_3_, the antisite-stabilised variant hence falls into the compositional range consistent with our CALPHAD-based phase diagram as well as with the previous reports.

## 1. Introduction

Intermetallics containing Al—the most abundant metal in the Earth’s solid crust—and transition metals (TMs) exhibit resistance to oxidation and corrosion, considerable hardness, low density, and high melting points [1,2]. Owing to their low diffusivity in Al, TMs perfectly meet the requirements for long-term strengthening of aluminides at elevated temperatures, which is an important prerequisite for applications in high-performance automobiles, railway cars, airplanes, spacecrafts or light ships. Among transition metals, Nb is a promising alloying element to improve wear resistance and strength of Al compounds [3]. The difference in crystal structure (bcc-Nb, fcc-Al) together with the largely disparate melting points (2743 and 942 K for Nb and Al, respectively), nevertheless, makes niobium aluminide compounds challenging to prepare as bulk. In terms of thin films, physical vapour deposition (PVD) techniques, such as magnetron sputter deposition or cathodic arc deposition (CAD), allow for synthesis of materials from the vapour phase [4]. Using such plasma-based deposition processes, phases not accessible by bulk methods, e.g., metastable phases, can be synthesised. However, the plasma properties and hence the thin film growth conditions are largely affected by the presence of the cathode surface, which is in a direct contact with plasma. In particular, in the case of CAD the formation of intermetallic phases on the cathode surface due to the exposure to the plasma affects the plasma properties, such as ion charge states and energies [5,6,7].

The published Al–Nb phase diagrams [3,8,9,10,11,12] show three single-phase fields corresponding to intermetallic phases: the body-centred tetragonal (bct) *ε*-Al_3_Nb (I4/mmm, #139, DO_22_), the tetragonal *σ*-AlNb_2_ (P4_2_/mnm, #136), and the cubic *δ*-AlNb_3_ (Pm3n, #223, A15) phase. According to Jorda et al. [3], the δ-phase is promising as a superconductor with transition temperature $T_{c}=18.8\;K$ [13]. Wen et al. [14] have recently correlated superconducting properties of *δ*-AlNb_3_ with kinetic mechanism of its phase formation. The authors further stated that deviations from the exact 1:3 Al-to-Nb stoichiometry leads to a significant decrease of $T_{c}$. Intriguingly, the reported compositional window for *δ*-AlNb_3_ does not contain the nominal $x_{\mathrm{Nb}}=0.75$ composition [12]. He et al. [15] have recently pointed out further discrepancies regarding the homogeneity ranges and melting points of Al–Nb compounds reported in the literature and proposed a new thermodynamic description of the system. Still, also their phase diagram calculated for temperatures 500–2500 K features for AlNb_3_ stability range *x*_Nb_ = 0.79–0.84, which is a somewhat surprising span for a phase with nominally $x_{\mathrm{Nb}}=0.75$.

Applying *ab initio* and CALPHAD calculations, we aim to clarify the (meta)stability and stoichiometry of δ-AlNb_3_ as well as to extend the existing phase diagrams to lower temperatures. According to our DFT predictions, Al_3_Nb and AlNb_2_ are the only stable phases at 0 K, while the lowest-energy δ-AlNb_3_ crystallographic variant still appears above the convex hull line (connecting all stable structures in the Al–Nb system). Moreover, phonon spectrum of δ-AlNb_3_ shows imaginary phonon frequencies from 0 up to 1500 K, which disproves any temperature-induced dynamical stabilisation. Yet this phase is present in both our CALPHAD-based phase diagram and XRD patterns recorded for powder-metallurgically produced arc cathodes. We show that the stability of AlNb_3_ is conditioned by the presence of point defects, which further allow to reach the experimentally reported higher Nb contents.

## 2. Modelling and Experimental Details

The phase diagram of Al–Nb binary system was constructed using CALculation of PHAse Diagram (CALPHAD) method [16]. Thermodynamic description proposed by Witusiewicz et al. [12] was adopted, whereas the parameters for pure Al were slightly modified. Importantly, as Gibbs free energies for the stable and metastable structures of the pure elements were taken from the SGTE database [17], the calculated phase diagram is valid only above 298.15 K.

To access even lower temperatures, Density Functional Theory (DFT) calculations were performed using the Vienna Ab-initio Simulation Package (VASP) [18,19] together with plane-wave projector augmented wave (PAW) pseudopotentials [20] and the Perdew-Burke-Ernzerhof generalized gradient approximation [21] for the exchange and correlation effects. The plane-wave cutoff energy of 600 eV and the *k*-vector sampling of the Brillouin zone ensured a total energy accuracy of 10^−3^ eV/at. or better. The binary Al_3_Nb, AlNb_2_, and AlNb_3_, were assumed to adopt the bct DO_22_ (I4/mmm, #139, ε), tetragonal D8_*b*_ (P4_2_/mnm, #136, σ), and cubic A15-type (Pm3n, #223, δ) structure, respectively. Equilibrium lattice parameters were determined by fitting the energy *vs.* volume data with the Birch-Murnaghan equation of state [22]. Additional structural candidates for low-energy Al–Nb intermetallics were predicted employing first-principles evolutionary algorithms as implemented in the USPEX (Universal Structure Predictor: Evolutionary Xtallography) code [23,24,25]. In order to reveal Al_1−*x*_Nb_*x*_ structures with different Nb contents, we performed two USPEX runs in a variable-composition mode with 4–16 and 16–32 atoms in the simulation cell, respectively, with 150 individuals in the initial generation and 80 in the subsequent ones. To compare relative chemical stability of the predicted compounds, energy of formation, $E_{f}$, was calculated according to

(1) $$E_{f}=\frac{1}{\sum_{s}n_{s}}\left(E_{\mathrm{tot}}-\sum_{s}n_{s}\mu_{s}\right),$$

where $E_{\mathrm{tot}}$ is the total energy of the simulation cell, $n_{s}$ and $\mu_{s}$ are the number of atoms and the chemical potential, respectively, of a species s={Al,Nb}. The total energies per atom of fcc-Al and bcc-Nb were conventionally adopted for the $\mu_{\mathrm{Al}}$ and $\mu_{\mathrm{Nb}}$ chemical potentials, respectively. To identify the stable compounds, the convex hull was constructed by connecting the local minima in the $E_{f}$ vs. composition plot. At compositions with a potentially stable or nearly stable structures (i.e., lying on or closely above the convex hull), fixed-composition USPEX runs were performed with variable number of formula units from 1 up to 10. Furthermore, point defects (vacancies and antisites) were distributed in the *δ*-AlNb_3_ in either ordered, or disordered manner. The former configuration will be described later (Figure 5 and the corresponding discussion), while the latter was achieved by employing the Special Quasi-random Structure (SQS) method [26]. To verify whether or not the proposed structures satisfy conditions for dynamical stability, phonon dispersion curves throughout the Brillouin zone and phonon density of states were analysed employing the finite-displacement approach as implemented in the PHONOPY package [27]. Furthermore, we employed the self-consistent *ab initio* lattice dynamical method (SCAILD) [28] which allows to simulate temperature dependent phonon dispersion curves and densities of states. Elastic properties for selected structural candidates were evaluated by applying the stress-strain method [29,30], which yields the fourth-rank elastic tensor from the Hooke’s law, based on the predefined strains and the computed stresses. Using the Voigt’s notation, the obtained fourth-rank elastic tensors were projected onto 6 × 6 elastic matrices, imposing the symmetry of the equilibrium simulation cells [31]. The polycrystalline bulk, *B*, shear, *E*, and Young’s moduli, *E*, were evaluated using the standard formulae, see e.g., Supplemental material for Ref. [32].

On the experimental side, the AlNb_3_ cathode was produced by powder-metallurgical methods and purchased from Plansee Composite Materials GmbH in Lechbruck am See, Germany. The phases prevalent in the cathode were structurally characterised by means of X-ray diffraction (XRD) using a Bruker-AXS D8 Advance. diffractometer equipped with Cu-K*α* radiation and parallel optics. The diffractogram was recorded at 2θ angles ranging from 10 to 120° with a step size of 0.035° and a dwell time of 2s per step. For the determination of the lattice parameters by means of a Rietveld refinement, we applied the commercial software package TOPAS supplied by Bruker AXS, USA. Furthermore, the elemental composition of the cathode was measured by energy-dispersive X-ray spectroscopy (EDX) using an Oxford Instruments INCA EDX system which was attached to a Zeiss EVO 50 scanning electron microscope (SEM).

## 3. Results and Discussion

### 3.1. Al–Nb Phase Diagram down to 0 K

**Figure 1:**
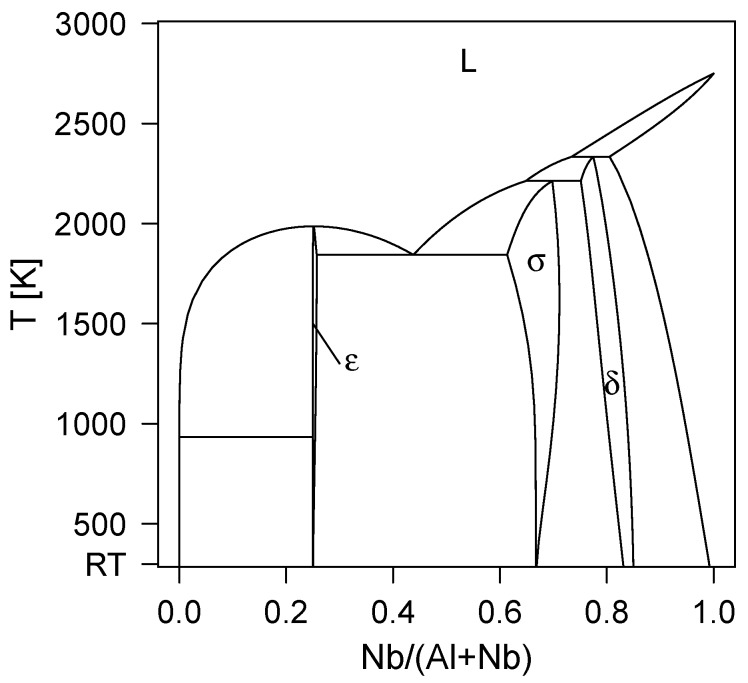
CALPHAD-based phase diagram of the binary Al–Nb system from room temperature (RT) up to 3000 K showing the *ε*-Al_3_Nb, *σ*-AlNb_2_, and *δ*-AlNb_3_ phase.

In accordance to the previous thermodynamic assessments of Al–Nb system [9,11,12,15], our CALPHAD-based phase diagram, Figure 1, shows *ε*-Al_3_Nb, *σ*-AlNb_2_, and *δ*-AlNb_3_ as stable phases. The stability range of *δ*-AlNb_3_ turns out to be peculiar in the same way as for the previous CALPHAD models, i.e., does not contain the nominal $x_{\mathrm{Nb}}=0.75$ composition, but actually goes far beyond that—up to about 82–84 at.% of Nb—when temperature decreases down to room temperature, 298.15 K.

**Figure 2:**
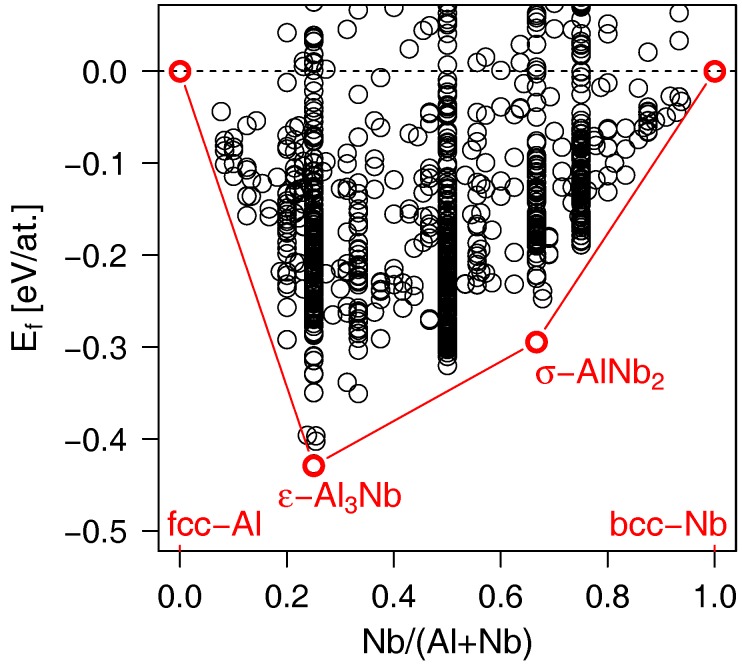
Energy of formation, $E_{f}$, of various Al_1−*x*_Nb_*x*_ structures predicted by first-principles evolutionary algorithm USPEX as a function of the Nb content. The convex hull line (red) connects the stable phases.

In order to investigate phase stability at even lower temperatures, in particular, to probe the extreme 0 K case, we employed *ab initio* calculations. No assumption on either stoichiometries, or crystal structures of the stable Al–Nb intermetallics was made. Instead, various Al_1−*x*_Nb_*x*_ structural candidates were generated by the evolutionary algorithm USPEX and subsequenty relaxed by DFT. The measure of their chemical stability was quantified by calculating formation energy, $E_{f}$, which is presented in Figure 2 as a function of the Nb content. To identify the stable compounds, a convex hull is constructed by connecting the local minima in the $E_{f}$ vs. composition data. The structures lying above the convex hull line and yielding negative $E_{f}$ values are deemed metastable. As expected, the tetragonal *ε*-Al_3_Nb and *σ*-AlNb_2_ phases have been correctly reproduced and found on the convex hull (i.e., stable). Nonetheless, the cubic *δ*-AlNb_3_—yielding the lowest $E_{f}$ out of all the predicted polymorphs at the $x_{\mathrm{Nb}}=0.75$ composition—appears about ∼0.03 eV/at. above the convex hull. Such finding disagrees with the previous calculations by Colinet et al. [10] employing the linear-muffin-tin-orbital (LMTO) method in the full potential (FP) approach and reporting *δ*-AlNb_3_ on the convex hull line, i.e., as a stable phase, exactly at $x_{\mathrm{Nb}}=0.75$. Unfortunately, we were unable to reproduce their convex hull irrespectively of various GGA-DFT or LDA-DFT flavours of Al and Nb pseudopotentials used in the simulation.

Contradictory to the published as well as our own (Figure 1) phase diagrams, no stable compound (i.e., lying on the convex hull) is predicted within the critical compositional window *x*_Nb_ = 0.79–0.84. Here we note that evolutionary algorithms do not guarantee finding the ground state structure, since the search space is infinite in principle, but a finite (user-controlled) number of generations is produced. Naturally, USPEX performs the best for small systems. If a unit cell is so large that it exceeds the (user-controlled) maximum number of atoms allowed in the simulation box, it can never be found. For that reason, additional variable-composition evolutionary search was carried out in the *x*_Nb_ = 0.7–0.9 range with 32–64 atoms in the unit cell, which was, however, computationally demanding and converged very slowly. Being aware of the above limitations, so far we could not disprove the existence of *δ*-AlNb_3_ or any other Al_1−*x*_Nb_*x*_ structural variant lying within the critical *x*_Nb_ = 0.79–0.84 compositional window.

### 3.2. Experimental Observation of AlNb_3_

**Figure 3:**
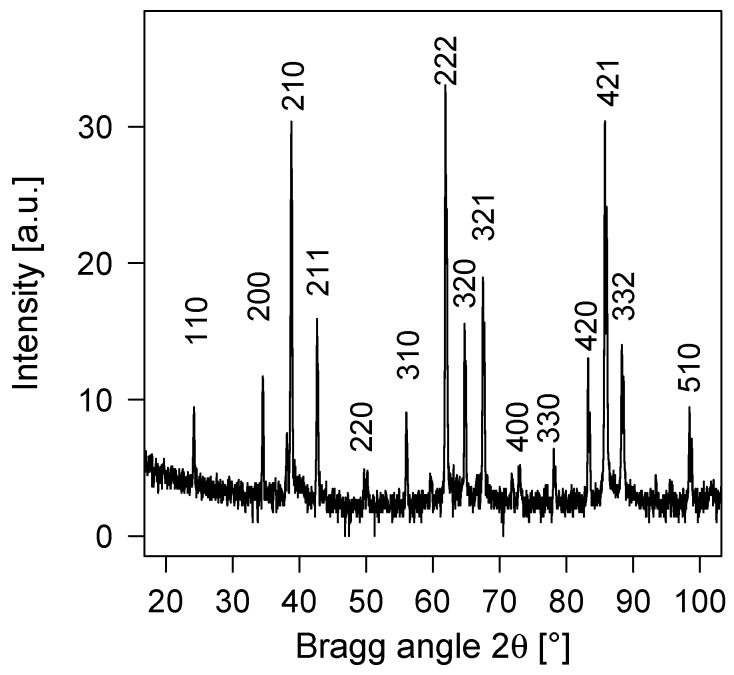
Indexed XRD pattern recorded for AlNb_3_ cathodes.

In parallel to the initial calculations, we analysed a cathode with nominal composition $x_{\mathrm{Nb}}=0.75$ available in our laboratory. The resulting X-ray diffractogram recorded in the Bragg-Brentano geometry, is shown in Figure 3. The peaks were labelled using our own DFT-optimised δ-AlNb_3_ (lying above the convex hull, cf. Figure 2), thus clearly demonstrating existence of this phase in experiment. The lattice constant determined through Rietveld refinement, $a_{\exp}=5.188$ Å, is in a good agreement with the DFT calculated value (a=5.1956 Å). The small offset is a well-known overestimation of the lattice constants by the GGA-DFT method. According to the elemental composition obtained by EDX, the chemistry of our sample is Al_0.22_Nb_0.78_, i.e., the sample shows a slight Nb overstoichiometry (as compared to the nominal 75 at.% of Nb). Therefore, the experiment (Figure 3) and the phase diagram (Figure 1) are consistent with each other, pointing towards stability of the AlNb_3_ phase, however, our DFT calculations so far suggest the opposite. To clarify this intriguing disagreement, we now turn our attention to possible stabilisation mechanisms of the δ-phase.

### 3.3. Stabilisation Mechanism of δ-AlNb_3_

**Figure 4:**
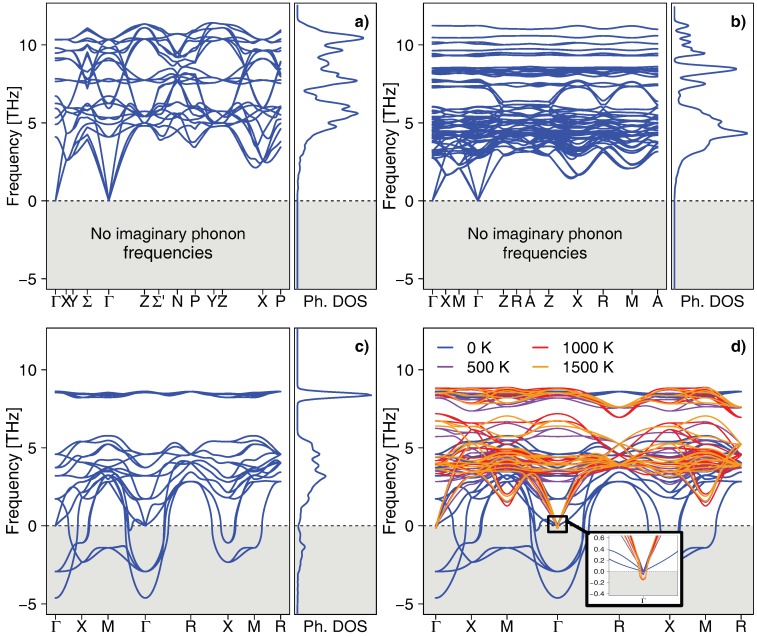
Phonon bandstructure and phonon DOS of the ε-Al_3_Nb (**a**), σ-AlNb_2_ (**b**), and δ-AlNb_3_ phase (**c**) at 0 K. The paths in the first Brilloiun zone were chosen based on the crystal symmetry considerations, as proposed by Setyawan and Curtarolo [34]. (**d**) Phonon bandstructure of δ-AlNb_3_ at elevated temperatures.

To shed light on the (meta)stability of the AlNb_3_ phase, lattice dynamics calculations were carried out. Figure 4 clearly demonstrates that while *ε*-Al_3_Nb and *σ*-AlNb_2_ satisfy conditions for dynamical stability, i.e., all their phonon frequencies are real, the *δ*-AlNb_3_ polymorph yields imaginary phonon modes (plotted as modes with negative frequencies) at, e.g., the Γ, *X*, and *M* points. Consequently, the atomic displacements corresponding to these soft phonon modes reduce the potential energy in the vicinity of the equilibrium atomic positions. Following some of these modes (not shown here), however, did not shift all the optical phonon branches from the imaginary to real frequencies, i.e., did not lead to a vibrationally stable structure. Additionally, evaluating phonon density of states (DOS) and phonon bandstructure of *δ*-AlNb_3_ under compression/tension by setting the cubic lattice parameter below/above the equilibrium value (not shown here) also did not lead to any stabilisation effect.

Despite the lack of dynamical stability at 0 K, the soft phonon modes might still disappear at higher temperatures, particularly when bearing in mind that the formation energy of *δ*-AlNb_3_ is only ≈0.03 eV/at. above the convex hull line (cf. Figure 2). Such temperature-induced stabilisation has been previously demonstrated, e.g., for the B2-NiTi phase [33] using the self-consistent *ab initio* lattice dynamical approach. We therefore employ the same approach also for the δ-phase of AlNb_3_, exploring temperatures from 0 up to 1500 K. Figure 4d indicates that despite reducing the imaginary phonon modes noticeably, the effect of elevated temperature is not sufficient to stabilise *δ*-AlNb_3_ due to the remaining instabilities at the Γ point. In contrast to the 0 K case, elevated temperature leads to a significant reduction of the gap of forbidden frequencies at ≈6–8 THz. Bandstructure in this particular frequency range, however, does not saturate but changes significantly at each temperature considered (500, 1000, and 1500 K).

### 3.4. Antisites Leading to the Off-Stoichiometry

**Figure 5:**
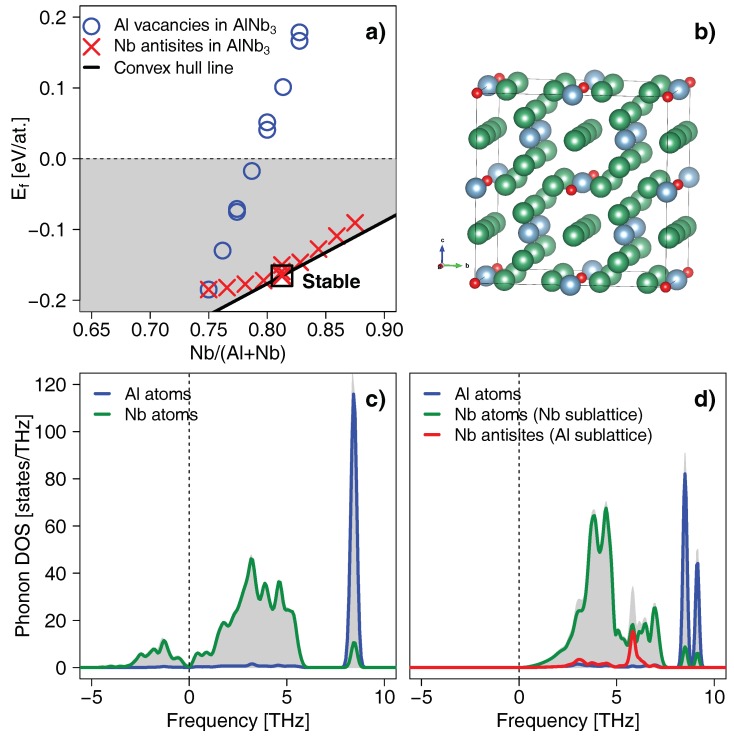
(**a**) Formation energy of the δ-AlNb_3_ phase containing Al vacancies (circles) or Nb antisites (crosses) as a function of the Nb content. The black solid line shows the convex hull, while the shaded area denotes region for the chemically stable compounds. (**b**) The δ-Al_0.19_Nb_0.81_ structural variant with ordered Nb antisites lying on the convex hull. The blue, green, and red spheres denote Al, Nb, and Nb antisite atoms, respectively. The phonon DOS of the defect-free δ-AlNb_3_ (**c**) compared to that of the δ-Al_0.19_Nb_0.81_ with ordered Nb antisites (**d**). The grey shaded area represents the total phonon DOS, while the solid lines show the partial contribution from Al and Nb atoms.

Excluding pressure and temperature induced dynamical stabilisation of the *δ*-AlNb_3_ phase, we further investigate the impact of point defects, which have been identified as key contributors to the stabilisation of numerous nitride [35,36,37], oxide [38], and carbide [39,40] systems. Since we wish to slightly alter composition to reach the experimental stability range of the δ-phase, the desirable defects are those which change stoichiometry. Conveniently, Al vacancies and Nb antisites (i.e., Nb atoms occupying some of the Al lattice sites) allow to shift the Nb content above the nominal 75 at.%. Figure 5a presents formation energies of the defected *δ*-AlNb_3_ phase as a function of the Nb content. Clearly, vacancies on the Al sublattice lead to a massive increase of $E_{f}$, hence destabilise the structure. Unlike that, $E_{f}$ increases only slightly upon populating some of the Al sites by Nb, i.e., by introducing antisite defects, and falls exactly on the convex hull line when the Nb content reaches 81.5 at.%. Our DFT calculations further suggest that the Nb antisite defects tend to order, as the disordered configurations (with antisites generated randomly according to the SQS method, cf. the Methodology section) result in slightly higher formation energies (about ∼0.01 eV/at.). The ordered distribution of Nb antisites in δ-Al_0.19_Nb_0.81_ can be described in a way that the Al sublattice forms an fcc-like structure (the structural model is presented in Figure 5b). Such ordered configuration yields lattice parameter of 5.215 Å, which exceeds the 5.196 Å of the defect-free AlNb_3_. Nevertheless, both these values can be regarded as in agreement with the experimentally estimated lattice parameter of 5.188 Å. Importantly, the corresponding phonon DOS of the ordered δ-Al_0.19_Nb_0.81_ does no longer show imaginary phonon frequencies, which clearly underpins the fact that $E_{f}$ of this phase falls on the convex hull (Figure 5a). This structural variant is thus dynamically stable. Noteworthy, the relatively large spread of structural parameters, 5.196–5.251 Å, predicted for various antisite distributions from ordered to disordered, overlaps with the 5.196 Å of the defect-free *δ*-AlNb_3_. Since the energetic difference of ∼0.01 eV/at. between the ordered the disordered structures becomes negligible at higher temperatures, the defect-free and antisite-containing δ-phase can be easily exchangeable in experiments.

### 3.5. Convex Hull at Finite Temperatures

**Figure 6:**
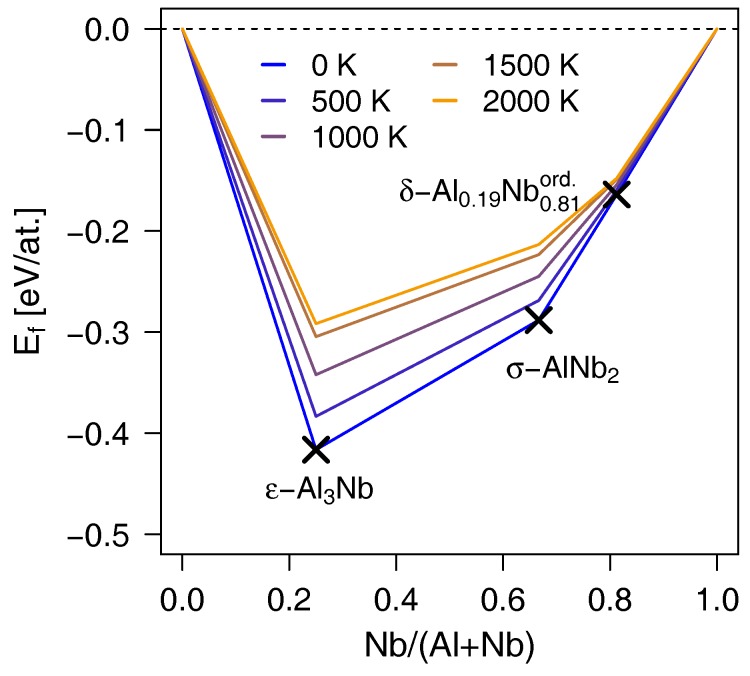
Energetics of the stable Al–Nb intermetallics—forming the convex hull—as a function of temperature.

To verify if the proposed δ-Al_0.19_Nb_0.81_ is stable also at higher temperatures, we calculated Helmholtz free energies of the structures forming the convex hull as a function of temperature, *T*, from 0 up to 2000 K. As the ordered δ-Al_0.19_Nb_0.81_ variant posseses higher symmetry and hence, is computationally easier to handle than the disordered polymorphs, it was considered for investigations of high temperature stability. Nonetheless, we note that Helmholtz free energies of the disordered configurations would have additional contribution form the mixing entropy, helping to stabilise them with respect to the ordered phase. Figure 6 displays the calculated temperature evolution of the Al–Nb convex hull. Interestingly, chemical stability of the δ-Al_0.19_Nb_0.81_ structure is only little affected by *T*, in contrast to the ε-Al_3_Nb and σ-AlNb_2_. This small dependence of the Helmholtz free energy on temperature causes that δ-Al_0.19_Nb_0.81_ always falls on the convex hull line and not above it. Hence, despite the overall shape of the convex hull changes noticeably, the stable compounds do not differ from the 0 K case and the antisite-stabilised structure is predicted to form also at higher temperatures.

### 3.6. Mechanical Properties of Stable Phases

Table 1 summarises structural and elastic data calculated for the stable Al_1−*x*_Nb_*x*_ phases, including the newly proposed antisite-stabilised ordered δ-Al_0.19_Nb_0.81_. We note that vibrational instability of the δ-AlNb_3_ phase at 0 K is related to optical phonons (cf. Figure 4), therefore, cannot be revealed by evaluating elastic constants. Indeed, elastic constants for both δ-Al_0.19_Nb_0.81_ (Table 1) and δ-AlNb_3_ (not shown here) satisfy conditions for mechanical stability [41]. Comparing elastic response of all the three stable Al_1−*x*_Nb_*x*_ intermetallic phases, i.e., for x={0.25,0.67,0.81}, our calculations suggest that an increase of *x* leads to a slight increase of *B* (from 134 to 165 GPa), whereas both *G* and *E* decrease (from 102 to 62 GPa and from 242 to 166 GPa, respectively). While the ratio $E_{100}$/$E_{111}=0.82$ obtained for ϵ-Al_3_Nb indicates that the (111) crystallographic direction is stiffer as compared to (100), this is not true for σ-AlNb_2_ and δ-Al_0.19_Nb_0.81_ yielding $E_{100}$/$E_{111}$ value of 1.16 and 1.36, respectively. Furthermore, both B/G and ν=(3B−2G)/(6B+2G) values, conventionally employed as relative empirical estimates of ductility [42,43], are predicted to increase with Nb content. We therefore envision that the order of ductility is Al_3_Nb < AlNb_2_ < Al_0.19_Nb_0.81_.

## 4. Conclusions

Stability of Al_1−*x*_Nb_*x*_ intermetallics was re-assessed combining theoretical (CALPHAD, DFT, evolutionary algorithms) and experimental (XRD, EDX) approaches. In agreement with previous reports, our phase diagram (extended down to room temperature) showed ε-Al_3_Nb, σ-AlNb_2_, and δ-AlNb_3_ as stable, yielding a peculiar compositional window *x*_Nb_ = 0.79–0.84 for the AlNb_3_ phase. First-principles evolutionary algorithms at 0 K, however, only uncovered the ε-Al_3_Nb and σ-AlNb_2_ intermetallics as stable. The δ-AlNb_3_—clearly present in both our phase diagram and XRD patterns recorderd for Al_0.25_Nb_0.75_ arc cathode—was found dynamically unstable (featuring imaginary phonon frequencies in the first Brillouin zone). We propose that AlNb_3_ can be stabilised by populating 25% of Al lattice sites with Nb, i.e., by antisite defects formation. Importantly, the resulting off-stoichimetric δ-Al_0.19_Nb_0.81_ configuration satisfies conditions for dynamical stability, lies on the convex hull line from 0 K up to 2000 K, and falls within the experimental compositional window. Such structural model of δ-AlNb_3_ allows to explain a long-standing discrepancy between first principles-based predictions on one hand and experimental observations and thermodynamic assessments on the other hand, and hence brings new and more accurate insights into understanding structural stability of the Al–Nb system.

## Figures and Tables

**Table 1:** Lattice parameters of ϵ-Al_3_Nb, σ-AlNb_2_, and the ordered δ-Al_0.19_Nb_0.81_ together with the corresponding elastic constants ($C_{ij}$ in GPa), the polycrystalline bulk (*B* in GPa), shear (*G* in GPa) and Youngs’s moduli (*E* in GPa). The B/G and the Poisson’s ratio (ν) represent ductility estimates, while the $E_{100}$/$E_{111}$ ratio between the Young’s moduli in the (100) and (111) direction relates to elastic isotropy.

	*a*	*c*	*C*_11_	*C*_12_	*C*_13_	*C*_33_	*C*_44_	B	G	E	B/G	*ν*	*E*_100_/*E*_111_
*ϵ*-Al_3_Nb	3.851	8.644	248	96	46	267	99	132	102	242	1.23	0.18	0.82
*σ*-AlNb_2_	5.188	9.978	278	98	100	284	74	159	81	208	1.96	0.28	1.16
*δ*-Al_0.19_Nb_0.81_	5.215		268	114			54	165	62	166	2.62	0.33	1.36
